# Dietary L-Arginine or N-Carbamylglutamate Alleviates Colonic Barrier Injury, Oxidative Stress, and Inflammation by Modulation of Intestinal Microbiota in Intrauterine Growth-Retarded Suckling Lambs

**DOI:** 10.3390/antiox11112251

**Published:** 2022-11-15

**Authors:** Hao Zhang, Yi Zheng, Xia Zha, Yi Ma, Xiaoyun Liu, Mabrouk Elsabagh, Hongrong Wang, Mengzhi Wang

**Affiliations:** 1 Laboratory of Metabolic Manipulation of Herbivorous Animal Nutrition, College of Animal Science and Technology, Yangzhou University, Yangzhou 225009, China; 2 Joint International Research Laboratory of Agriculture and Agri-Product Safety, The Ministry of Education of China, Yangzhou University, Yangzhou 225009, China; 3 Department of Animal Production and Technology, Faculty of Agricultural Sciences and Technologies, Niğde Ömer Halisdemir University, Nigde 51240, Turkey; 4 Department of Nutrition and Clinical Nutrition, Faculty of Veterinary Medicine, Kafrelsheikh University, Kafrelsheikh 33516, Egypt

**Keywords:** L-arginine, suckling lambs, intrauterine growth restriction, inflammation, colonic barrier function, N-carbamylglutamate

## Abstract

Our previous studies have revealed that dietary N-carbamylglutamate (NCG) and L-arginine (Arg) supplementation improves redox status and suppresses apoptosis in the colon of suckling Hu lambs with intrauterine growth retardation (IUGR). However, no studies have reported the function of Arg or NCG in the colonic microbial communities, barrier function, and inflammation in IUGR-suckling lambs. This work aimed to further investigate how dietary Arg or NCG influences the microbiota, barrier function, and inflammation in the colon of IUGR lambs. Forty-eight newborn Hu lambs of 7 d old were assigned to four treatment groups (*n* = 12 per group; six male, six female) as follows: CON (normal birth weight, 4.25 ± 0.14 kg), IUGR (3.01 ± 0.12 kg), IUGR + Arg (2.99 ± 0.13 kg), and IUGR + NCG (3.03 ± 0.11 kg). A total of 1% Arg or 0.1% NCG was supplemented in a basal diet of milk replacer, respectively. Lambs were fed the milk replacer for 21 d until 28 d after birth. Compared to the non-supplemented IUGR lambs, the transepithelial electrical resistance (TER) was higher, while fluorescein isothiocyanate dextran 4 kDa (FD4) was lower in the colon of the NCG- or Arg-supplemented IUGR lambs (*p* < 0.05). The IUGR lambs exhibited higher (*p* < 0.05) colonic interleukin (IL)-6, IL-1β, tumor necrosis factor (TNF)-α, reactive oxygen species (ROS), and malondialdehyde (MDA) levels than the CON lambs; the detrimental effects of IUGR on colonic proinflammatory cytokine concentrations and redox status were counteracted by dietary Arg or NCG supplementation. Both IUGR + Arg and IUGR + NCG lambs exhibited an elevated protein and mRNA expression of Occludin, Claudin-1, and zonula occludens-1 (ZO-1) compared to the IUGR lambs (*p* < 0.05). Additionally, the lipopolysaccharide (LPS) concentration was decreased while the levels of acetate, butyrate, and propionate were increased in IUGR + Arg and IUGR + NCG lambs compared to the IUGR lambs (*p* < 0.05). The relative abundance of *Clostridium*, *Lactobacillus*, and *Streptococcus* was lower in the colonic mucosa of the IUGR lambs than in the CON lambs (*p* < 0.05) but was restored upon the dietary supplementation of Arg or NCG to the IUGR lambs (*p* < 0.05). Both Arg and NCG can alleviate colonic barrier injury, oxidative stress (OS), and inflammation by the modulation of colonic microbiota in IUGR-suckling lambs. This work contributes to improving knowledge about the crosstalk among gut microbiota, immunity, OS, and barrier function and emphasizes the potential of Arg or NCG in health enhancement as feed additives in the early life nutrition of ruminants.

## 1. Introduction

Insufficient fetal nutrition or maternal malnutrition-induced placental dysfunctions predispose intrauterine growth retardation (IUGR) when a baby’s birth weight is less than the 10th percentile [1]. From the epidemiological perspective, IUGR is also a possible contributor to gastrointestinal conditions, such as irritable bowel syndrome [2], inflammatory bowel disease (IBD) [3], and colorectal cancer (CCR) [4]. Furthermore, according to previous animal experimentation, the intestinal barrier functions were altered permanently by IUGR, including the decreased quantity and function of Paneth and goblet cells, a more permeable colon, as well as impaired growth, differentiation, and death of cells [5]. Clarifying the mechanisms underlying these dysfunctions is imperative to identify potential preventive dietary interventions. In this study, we hypothesized that colonic mucosal barrier disruption, oxidative stress (OS), inflammatory response, and microbiota disorder could be responsible for intestinal frailty in lambs born with IUGR.

The colon, as an intestinal section, has the highest microbial community and vigorous metabolism of microorganisms [6]. The mucosal microbiota of the colon promotes the growth of local immune cells and educates the host immunity [7]. The intestinal microbiota is involved in the childhood host metabolism, gastrointestinal tract (GIT) functions, immunity evolution, as well as cellular growth and differentiation [8]. In addition to altered birth weight, the gut microbiota composition can be disturbed during the intestinal development stage [9]. Besides, widely diverse metabolites are synthesized by the intestinal microbiota, starting from the dietary precursors, whose favorable role in energy metabolism and enterocyte functions has been acknowledged [10]. The existing literature has regarded nutritional regulation as an efficient way to modulate gut microbiota [11]. However, there have been rare investigations about how the mucosal microbiota of the colon is affected by nutritional interventions in newborn lambs, which necessitates more investigations.

As an essential amino acid for young animals, L-arginine (Arg) enhances the performance of growth, the release of digestive enzymes, and the expression of nutrient transporters [12]. It also enhances the antioxidant state, immunity, and gut barrier functions [13]. The role of Arg in modulating the metabolism and composition of microbiota has been previously reported. Arg-supplemented dentifrice could positively regulate the oral microbiota of individuals with dental decay, as manifested by the enhanced abundance of alkali-producing microbes and the diminished content of acid-forming microbes [14]. Supplemental rumen-protected Arg affected sika deer metabolism and their gut microbiota [15]. N-carbamylglutamate (NCG), a stable synthetic derivative of glutamic acid [16], effectively activates carbamyl phosphate synthase-1, a key enzyme in Arg synthesis in enterocytes [17]. Dietary NCG increased the body weight (BW) gain in weaned piglets partly by favorably affecting intestinal growth and integrity [18]. Dietary NCG and Arg supplementation improves redox status and suppresses apoptosis in the colon of IUGR-suckling lambs [19]. Yet, the role of Arg or NCG in colonic microbiota modulation has never been reported in IUGR-suckling lambs.

To this end, the objective of this study was to clarify how the dietary Arg or NCG supplementation affects colonic barrier function, inflammation, and colonic microbiota in IUGR lambs.

## 2. Materials and Methods

All animal procedures were in accord with and approved by the Animal Ethical Committee of Yangzhou University (SXXY 2015-0054). 

### 2.1. Animals and Treatments 

Suckling Hu lambs were identified as IUGR when the birth weights were more than two standard deviations (SD) below the mean. On the other side, the birth weights of suckling Hu lambs were deemed normal birth weight (NBW) when their values approximated the mean litter birth weight, that is, within 0.5 SD [20]. On d 7 after birth, 12 and 36 newborn Hu lambs weighing 4.25 ± 0.14 kg (NBW) and 3.01 ± 0.12 kg (IUGR), respectively, were selected from a cohort of 432 twin lambs (4.16 ± 0.15 kg) at the Jiangyan Experimental Station (Taizhou, Jiangsu, China). After weaning and isolating the lambs from their dams at 7 d of age, they were assigned into one of 4 groups (*n* = 12 per group) in triplicates with 2 males and 2 females per replicate, depending on their initial BW. The 4 groups were named the CON (4.25 ± 0.14 kg), NBW lambs fed milk replacer (MR); the IUGR (3.01 ± 0.12 kg), IUGR lambs fed MR; the IUGR + Arg (2.99 ± 0.13 kg), IUGR lambs fed MR supplemented with 1% Arg; and the IUGR + NCG (3.03 ± 0.11 kg) group, IUGR lambs fed MR supplemented with 0.1% NCG, respectively [21]. During a 21-d-long trial (until 28th d postpartum), the lambs in every replicate were kept in an indoor pen (4 × 1 m^2^) and received a basal MR diet (iso-caloric and iso-nitrogenous; Supplementary Table S1) satisfying the nutritional requirements for suckling lambs [22] with free access to fresh water. For nitrogen equilibration, the L-alanine from Ajinomoto in China’s Beijing was used. The NCG (purity: 97%) used was the product of Sigma–Aldrich (Louis, MI, USA), while Arg was the product of Ajinomoto (Beijing, China). The dose of NCG (0.1%) and Arg (1%) was determined based on previous trials of lambs [23] and piglets [24]. The MR feeding rate was adjusted every 10 d at a rate of 2% of the live body weight of each lamb. Hot water was used to dissolve the MR before feeding, which was made into a 16.67% DM solution (40 °C) and was offered to the lambs three times a day (07:00, 13:00, and 19:00). To avoid confounding variables, such as daily management and handling, the MR was fed by veteran farm staff. The daily consumption of MR was calculated as the difference between the offered and the rejected MR amount for each lamb. For the daily estimation of MR’s average dry matter intake (ADMI), the average daily consumption of MR was multiplied by its dry matter (DM) content (%). The lambs were weighed for their initial (7 d of age) and final (28 d of age) BW. The results of the growth performance have been published elsewhere [25]. 

### 2.2. Chemical Analyses of MR

The MR samples were analyzed for the ash, DM, crude protein (CP), ether extract (EE), calcium (Ca), and total phosphorus (TP) (methods 942.05, 930.15, 990.02, 920.39, 968.08, and 965.17, respectively, [26]). The gross energy (GE) was measured using a bomb calorimeter (C200; IKA Works Inc., Staufen, Germany). The AA profile was measured by reverse-phase HPLC (HP1100; Agilent) using norleucine as the internal standard according to published methods [27].

### 2.3. Tissue Sample Collection 

On the 7th and 28th days of experimentation, the weights of the suckling lambs were recorded. At 08:00 on day 28, after an overnight fast, all the lambs were anaesthetized with an intravenous injection of sodium pentobarbital (15 mg/kg BW), and the colonic digesta was sampled immediately. The colonic digesta was diluted with distilled water (double volume) and was centrifuged at 2000× *g* for 10 min to separate the supernatants, which were stored at −20 °C for the measurement of volatile fatty acids (VFAs) according to Qin [28], as will be described later. In the meantime, the colonic mucosa was scraped with sterile slides and was kept at −80 °C in liquid nitrogen for the later extraction of DNA. In parallel, the intact colonic tissue samples were sampled and washed using ice-cold saline buffered with phosphate and were sectioned into 0.4 cm × 0.4 cm pieces and preserved at −80 °C for later analytical use [29].

### 2.4. Determination of Epithelial Tight Junction Permeability of the Colon

The transepithelial electric resistance (TER) (Ω·cm^2^) and paracellular fluorescein isothiocyanate dextran 4 kDa (FD4) flux were measured using the Using chamber following a previous study protocol [30]. A microplate fluorescence reader (FLx800, Bio-Tek, Winooski, VT, USA) was used to determine the FD4 fluxes at the serosa side [31].

### 2.5. Cytokine Analysis of the Colon Tissue

Commercially available kits were used to determine the levels of interleukin-1β (IL-1β) (BioSource/MED Probe, Camarillo, CA, USA), as well as interleukin-6 (IL-6) and tumor necrosis factor-α (TNF-α) (both from R&D Systems, Oxford, UK). The absorbance was measured at a wavelength of 450 nm using a synergy HT microplate reader (BioTek Instruments, Winooski, VT, USA), and the results were expressed as ng/g of the colonic tissue protein level [20].

### 2.6. Colonic VFAs and LPS Concentration

The VFAs (acetate, butyrate, isobutyrate, isovalerate, propionate, and valerate) in the colonic digesta were determined using gas chromatography (14B, Shimadzu, Kyoto, Japan) with a 30 m × 0.32 mm × 0.25 mm film-thick capillary column. The chromatography was run at temperatures of 180 °C, 110 °C, and 180 °C for the injection, column, and detector, respectively, using crotonic acid as an internal standard [32]. The detailed method of VFAs determination was described by Qin [28]. The lipopolysaccharide (LPS) content in the colonic digesta was assayed using an ELISA Kit (Cloud-Clone, Houston, TX, USA) [33].

### 2.7. mRNA Abundance

The total RNA was extracted from 100 mg of colonic mucosa using Trizol (15596026; Thermo Fisher Scientific, Waltham, MA, USA), and both the RNA quality and concentration were assayed by spectrophotometry (NanoDrop ND-1000, Thermo Fisher Scientific) [34]. Afterward, RNase-Free DNase (M6101; Promega, Madison, WI, USA) was used to treat the total RNA (2 mg) and to reversely transcribe them according to the manufacturer’s protocol. Real-time PCR was accomplished in Mx3000P (Stratagene, CA, USA) with 2 µL of 1:20 (*v*/*v*) cDNA dilution. *β*-actin, which was irrelevant to experimental factors, was selected as the reference gene. The primers used for the gene expression are presented in Supplementary Table S2, all of which were synthesized by The Generay Biotech in China’s Shanghai. Real-time PCR findings were analyzed by the 2^−ΔΔCt^ approach [35], while the mRNA levels of genes were represented as fold change relative to the mean of the control group.

### 2.8. Western Blotting

The total proteins in the colonic tissue were extracted using a commercial kit (Beyotime Biotechnology, Jiangsu, China) and were homogenized according to the manufacturer’s protocol. Afterward, the bicinchoninic acid assaying kit (Pierce, Rockford, IL, USA) was used to determine the protein concentration. The primary antibodies used in the present study were as follows: anti-ZO-1, anti-occludin, and anti-claudin-1 were all 1:1000 dilutions from Abcam, while anti-β-actin was 1:1500 dilutions from Santa Cruz. Afterward, a 45 min protein incubation was accomplished using the goat HRP-labeled anti-rabbit IgG secondary antibody (1:1000, Beyotime), and then visualization of signals proceeded via an enhanced chemiluminescence kit (Thermo Fisher Scientific, USA) with a subsequent imaging detector (Bio-Rad, Shanghai, China). The ImageJ program was used for data processing. The gray-value quantification was performed relative to the β-actin value and was represented against the control [36]. Every trial was replicated 6 times.

### 2.9. Bacterial DNA Extraction of Colonic Mucosa

The weight of the colonic mucosa used for DNA extraction was 0.25 g in total. After conducting the extraction procedure via a QIAamp Fast DNA Stool Mini Kit (Qiagen, Hilden, Germany), a Nanodrop 2000 spectrophotometer (Thermo Fisher Scientific, Waltham, MA, USA) was used for quantification. For the lysing efficiency improvement, a mixture of the mucosal sample, Inhibit EX buffer (1 mL), and 0.1 mm zirconium beads (100 mg) was homogenized with Mini-Beadbeater-1 (Biospec Products, Shanghai, China) in a centrifuge tube, which was then subjected to 5 min heating at 95 °C for improving the DNA production, followed by 1 min centrifugation. In a new centrifuge tube, the resulting supernatant was mixed with proteinase K (15 mL) for DNA purification purposes. The DNA specimens were preserved at −80 °C for later analytical use [37].

### 2.10. 16S rRNA Analysis of Colonic Mucosa-Associated Microbiota

Library preparations for next-generation sequencing (NGS) were performed at Novogene Inc. in China’s Nanjing, as well as the Illumina MiSeq sequencing. Regarding the primers used for amplifying the bacterial 16S ribosomal RNA (16S rRNA) V3–V4 region, they included 338F (5′-barcode-ACTCCTRCGGGAGGCAGCAG-3′) and 806R (5′-GGACTACCVGGGTATCTAAT-3′), which were about 470 bp in amplicon length. Adapter sequences were also available in these primers so that highly sophisticated libraries could be amplified evenly to prepare for the Illumina MiSeq-based downstream NGS. A thermal cycler (50 mL; Bio-Rad, US) was employed for accomplishing all PCRs, which contained a 5-fold FastPfu buffer (4 mL), DNA (10 ng), primers (10 ng each), Pfu polymerase (0.5 U), and 2.5 mM dNTPs (2 mL). The PCR amplification conditions were an initial step of 3 min at 95 °C, followed by 27 cycles of 30 s at 95 °C, 30 s at 55 °C, 45 s at 72 °C, and a final 10 min extension at 72 °C. A DNA gel Extraction Kit (AxyPrep, Axygen Biosciences, Union, CA, USA) was used for the purification of amplicons. Subsequently, a DNA Sample Prep Kit (TruSeq^TM^; TransGen Biotech, Beijing, China) was used to produce amplicon libraries according to the manufacturer’s protocol. Finally, an Illumina MiSeq platform was used for Paired-end sequencing (2 × 250 bp reads) according to the manufacturer’s protocol [38].

### 2.11. Sequence Processing and Analysis

The processing of data was accomplished via the QIIME package (ver. 1.70) [39]. The decoding of 16S rRNA reads was performed based on the sample-specific barcodes (6 bp), followed by the selection of superior-quality sequences. Sequences filtered from the raw data were deemed a superior quality when their length exceeded 200 bps, and the mean base quality score was above 25. At a 97% sequence similarity, UPARSE was employed to cluster sequences into the operational taxonomic units (OTUs) [40]. The identification of representative sequences was achieved for each OUTs by selecting the most abundant sequences, which were subsequently assigned to the Greengenes core set (ver. 13.5) separately [39]. Taxonomic allocation was made with the Bayesian classifier offered by the Ribosomal Database Project [41], through a comparison between the representative sequences in the Greengenes database and those in each cluster of OTUs [42], with the aid of PyNAST [38] using the default QIIME variables. For alpha diversity evaluation, the QIIME package (ver. 1.70) was used to estimate the Chao1 value, the abundance-based coverage estimator (ACE), the Simpson index, and the Shannon index. FastTree was used to construct a phylogenetic tree of representative sequences [43]. With the aid of the MOTHUR package (ver. 1.29), inter-sample dissimilarities were assessed through principal coordinate analysis (PCoA) based on the unweighted Unifrac distance in conjunction with molecular variance analysis (AMOVA) [44].

### 2.12. Statistical Analysis

Results are presented as the means and standard errors of the means (SEMs). The fixed effect of sex was incorporated into the original statistical model but was removed from the final model due to insignificance (*p* > 0.05). Accordingly, treatment was the only fixed variable. Data were evaluated by a one-way, completely randomized analysis of the variance of the GLM procedure of SPSS (ver. 20, IBM, Chicago, IL, USA). Tukey’s test was used to separate inter-treatment statistical differences. *p* values of ≤0.05 were regarded as significant.

## 3. Results

### 3.1. Mitochondrial Reactive Oxygen Species (ROS) Generation and Oxidative Status in the Colon

Compared to the CON group, the superoxide dismutase (SOD), glutathione peroxidase (GSH-Px), total antioxidant capacity (T-AOC), reduced glutathione (GSH) activities, and GSH/oxidized glutathione (GSSG) ratio were lower (*p* < 0.05), whereas the ROS production and levels of malondialdehyde (MDA), glutathione reductase (GR), hydrogen peroxide (H_2_O_2_), and protein carbonyl were higher (*p* < 0.05) in the colon of IUGR lambs (Supplementary Table S3). Relative to the IUGR lambs, colonic SOD, GSH-Px, T-AOC, GSH activities, and GSH/GSSG ratio was higher (*p* < 0.05), whereas the ROS production and levels of MDA, GR, H_2_O_2,_ and protein carbonyl were lower (*p* < 0.05) in the IUGR + Arg and IUGR + NCG lambs [19]. 

### 3.2. The Barrier Function of the Colon

Compared to the CON group, the TER was lower, and the FD4 was higher (*p* < 0.05) in the colon of the IUGR lambs (Table 1). Conversely, the IUGR + Arg and IUGR + NCG groups exhibited higher TER and lowered FD4 (*p* < 0.05) in the colon than the untreated IUGR lambs.

### 3.3. The Cytokine Concentrations in the Colonic Mucosa

Compared to other treatments, the IUGR lambs showed higher (*p* < 0.05) TNF-α, IL-6, and IL-1β levels in the colon (Table 2). The lowest proinflammatory cytokine levels were found in the CON lambs (*p* < 0.05). The detrimental impact of IUGR on the colonic levels of proinflammatory cytokine was counteracted in IUGR lambs supplemented with Arg or NCG (*p* < 0.05).

### 3.4. VFAs and LPS Concentrations in the Colonic Digesta

The levels of LPS were elevated (*p* < 0.05), while those of acetate, butyrate, isobutyrate, and propionate declined (*p* < 0.05) in the colonic digesta of IUGR lambs compared to the CON ones (Table 3). By contrast, the levels of LPS declined (*p* < 0.05) while levels of acetate, butyrate, isobutyrate, and propionate were elevated (*p* < 0.05) in the colonic digesta of IUGR + Arg and IUGR + NCG lambs compared to the IUGR lambs. 

### 3.5. Gene Expression

The colonic mRNA expressions of *TNF-α*, *IL-1β*, *IL-6*, *MyD88*, and *NF-κB* genes were higher (*p* < 0.05) in IUGR lambs than in the CON lambs but were lower (*p* < 0.05) in Arg-or NCG-supplemented IUGR lambs compared to the untreated IUGR ones (Table 4). For colonic mRNA levels of *ZO-1*, *Claudin-1*, and *Occludin*, their expressions were lower (*p* < 0.05) for the IUGR lambs than for the CON lambs but were increased (*p* < 0.05) upon the supplementation of Arg or NCG to IUGR lambs compared to the non-supplemented IUGR lambs.

### 3.6. The Protein Expression

**Figure 1:**
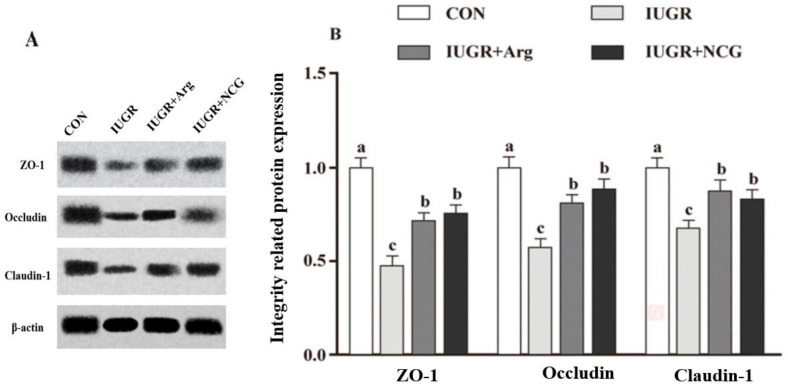
Effects of dietary Arg or NCG supplementation on the integrity-related protein expression in the colon of IUGR suckling lambs. Representative charts of Western blot results (**A**) and related protein expression of ZO-1, occludin, and claudin-1 (**B**) were obtained. The values are expressed as the mean with standard error represented by vertical bars (*n* = 12). CON: normal birth weight group given a control diet; IUGR: intrauterine-growth-retarded group given a control diet; IUGR + Arg: IUGR supplemented with 1% L-arginine; and IUGR + NCG: IUGR supplemented with 0.1% N-carbamylglutamate. ^a,b,c^ Mean values in the columns without a common letter differ (*p* < 0.05). ZO-1 = zonula occludens-1.

The Claudin-1, Occludin, and ZO-1 protein expression in the colonic mucosa declined (*p* < 0.05) in the IUGR lambs compared to the CON lambs (Figure 1). In contrast, the protein expression of these genes was elevated (*p* < 0.05) in the colonic mucosa of the IUGR + Arg and IUGR + NCG lambs than in the IUGR lambs. 

### 3.7. Diversity of the Colonic Mucosa-Associated Microbiota

**Figure 2:**
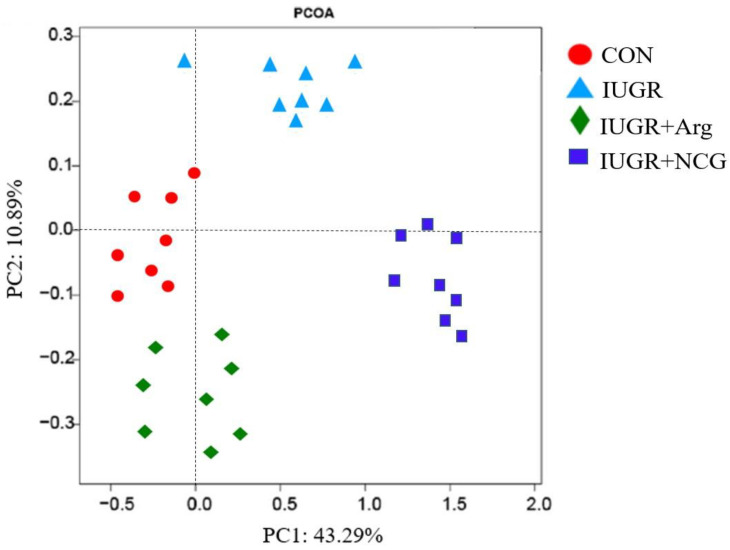
Principal component analysis (PCA) of bacterial community structures of the colonic microbiota in CON, IUGR, IUGR + Arg, and IUGR + NCG groups. CON: the normal birth weight group given a control diet; IUGR: the intrauterine-growth-retarded group given a control diet; IUGR + Arg: IUGR supplemented with 1% L-arginine; and IUGR + NCG: IUGR supplemented with 0.1% N-carbamylglutamate.

The IUGR lambs showed a reduced (*p* < 0.05) ACE value, Chao 1, OTU numbers, and Shannon index compared to the CON lambs when the similarity level was 97% (Table 5). On the contrary, the IUGR + Arg and IUGR + NCG lambs had an elevated (*p* < 0.05) ACE value, Chao 1, OTU numbers, and Shannon index compared to the IUGR lambs. Microbial composition in the colonic mucosa was distinct among the four groups, as revealed by the β-diversity analysis and the Bray–Curtis distance-based PCoA (Figure 2).

### 3.8. Colonic Mucosal Microbiota Composition

At the phylum level, the IUGR lambs exhibited higher (*p* < 0.05) relative abundances of *Bacteroidetes* and *Proteobacteria* and a lower (*p* < 0.05) relative abundance of *Firmicutes*, respectively, compared to the CON lambs (Table 6). As for the IUGR + Arg and IUGR + NCG lambs, they showed a higher (*p* < 0.05) relative abundance of *Firmicutes* and lower (*p* < 0.05) relative abundances of *Bacteroidetes* and *Proteobacteria*, respectively, compared to the IUGR lambs.

At the genus level, the colonic mucosa of IUGR lambs exhibited higher (*p* < 0.05) relative abundances of *Bacteroide*, *Staphylococcus*, *Pseudomonas*, and *Escherichia* compared to those in the CON lambs (Table 7). In contrast, the colonic mucosa of the IUGR + Arg and IUGR + NCG lambs experienced lower (*p* < 0.05) relative abundances of these genera compared to those in the IUGR lambs. Regarding the colonic mucosal relative abundances of *Clostridium*, *Lactobacillus*, and *Streptococcus*, their values were lower (*p* < 0.05) for the IUGR lambs than for the CON lambs but were higher (*p* < 0.05) for the IUGR + Arg and IUGR + NCG lambs than for the IUGR lambs.

## 4. Discussion

A 21 d dietary supplementation with NCG or Arg facilitated the gut development of IUGR lambs aged 7 days [23]. This effect was probably associated with the variations in colonic barrier functionality and inflammation by the modulation of colonic microbiota. The current study, therefore, was carried out to verify this assumption. It is unlikely that NCG or Arg supplements reach the colon and, thus, have a direct effect. This implies that the most likely mechanism is through the host. In suckling piglets, the majority of the citrulline synthesized in the enterocytes was locally converted into Arg [45]. From a mechanistic standpoint, the lower plasma citrulline concentration, a precursor of Arg, is indicative of intestinal dysfunctions in infants and adults [46]. In our previous study, plasma Arg and citrulline concentrations were increased in IUGR lambs in response to NCG and Arg supplementation [12], which is a further indication of an improvement in colonic functions.

The intestinal barrier prevents the mucosal tissue invasion of toxins, antigens, and pathogens and consequently exerts a vital role in equilibrating the gut environment [47]. The TER technique evaluates whether the intestinal epithelium is intact and permeable. Paracellular pathways are primarily responsible for the flux of FD4 across the intestinal epithelium. A damaged intestinal barrier can be identified by a decline in the TER, together with an elevation in the FD4 flux [48]. As a major constituent of the mechanical barrier of gut mucosa, tight junctions encompass plenty of proteins, such as Occludin, Claudin, and ZO-1, as well as junction adhesion molecules [49]. In this study, IUGR lambs supplemented with Arg or NCG exhibited higher TER and lowered FD4 flux in their colon compared to those of the untreated IUGR lambs. Furthermore, the ZO-1, Occludin, and Claudin-1 expressions were remarkably up-regulated in the Arg- or NCG- supplemented lambs at both mRNA and protein levels. Arg-induced increases in the protein expressions of Claudin-1 and ZO-1 were reported in an earlier study [50] and were associated with a decline in the FD4 and an elevation in TER in the LPS-treated ovine intestinal epithelial cells. Thus, the current presumption is that hazardous intra-luminal permeations (e.g., foreign microorganisms and their toxins) were avoided owing to the improved colonic barrier functionality by the supplementation of Arg or NCG to the IUGR suckling lambs.

Oxidative stress is widely recognized as a state of the imbalance of oxidation and antioxidation, which has been widely implicated in intestinal epithelium apoptosis [51]. An increased MDA level and decreased SOD and GSH-Px levels are generally considered markers of intestinal oxidative injury [52]. Our previous study has demonstrated that IUGR increased the colonic OS by decreasing the activities of antioxidant enzymes, such as GSH-Px and SOD, and increasing the ROS level [19]. Numerous studies have demonstrated that OS is associated with intestinal barrier dysfunction, low-grade inflammation, and various digestive tract diseases [53]. Therefore, dietary NCG and Arg supplementation in early-life nutrition alleviates the negative effects of OS damage and is crucial for the development of IUGR newborn lambs [19].

Enterocytes can offer a barrier against microbial infiltration via both congenital and adaptive immunity [54]. Toll-like receptors (TLRs), as major pattern recognition receptors present in the congenital immune defense system of organisms and exert a crucial role in the regulation of inflammatory reactions and related signaling [23]. As clarified by recent research concerning the TLR2 and TLR4 functions, the secretion of NF-κB from the I-kB/NF-κB complex and its intracellular transfer can be induced by the TLR2 and TLR4 up-regulation, which results in the secretion of associated inflammatory cytokines (e.g., IL-1β, IL-6, and TNF-α), and their involvement in the immune reactions against pathogenic bacteria [55]. The participation of the TLR4-MyD88-NF-κB axis in inflammation has been previously reported [56]. At a molecular level, the increased LPS from Gram-negative bacteria could be recognized by TLR4, which could then activate NF-κB and promote its nuclear translocation, thereby triggering the proinflammatory gene transcription [57]. Prominently elevated colonic mRNA abundances of proinflammatory cytokines were observed in the current study among suckling lambs with IUGR. The increased levels of IL-1β, IL-6, LPS, and TNF-α that were found in the colon of IUGR lambs are consistent with the gene expression findings. The dietary supplementation of Arg or NCG reduced the levels of the above-mentioned cytokines, as well as the expressions of *TNF-α*, *TLR-4*, *IL-1β*, *IL-6*, and *NF-κB* genes in the colon of IUGR suckling lambs. This suggests that the Arg and NCG can protect IUGR-suckling lambs from colonic inflammatory reactions.

As the primary energy source of enterocytes, VFAs can sustain intestinal cell functionality, modulate immunity, and mitigate diverse inflammatory diseases. In addition, VFAs are significantly associated with the metabolism of intestinal cells and the modulation of microbiota and genetic expressions [58]. There is a positive relationship between the butyrate-producing bacteria and the tight junction ZO-1 levels [59]. The levels of barrier-associated genes and short-chain fatty acid (SCFAs) receptors could be promoted by propionate, acetate, and branched-chain fatty acids [60]. In the present work, the levels of SCFAs (including acetate, butyrate, and propionate) were elevated in the colon of IUGR lambs by the dietary supplementation of Arg or NCG, which was probably conducive to making the colon more intact and lowering the proinflammatory cytokine levels.

Gut microbiota acts as an important bridge between host health and ambient substances. Gut microbiome dysbiosis is associated with increased colonic OS, barrier dysfunction, inflammatory responses, and systemic autoimmunity markers [61]. Lambs supplemented with Arg or NCG had more Firmicutes but less Escherichia and Proteobacteria than the IUGR lambs. Intestinal health would supposedly be boosted by relatively elevating the Firmicutes’ abundance [62]. The majority of SCFAs-generating bacteria are also Firmicutes family members. As the Firmicutes abundance increases, enhanced gut barrier functions and weakened inflammatory reactions and OS are observed [63]. Escherichia is a pathogenic genus in the Gram-negative Proteobacteria phylum, which also encompasses broadly diverse and other pathogenic genera [64]. The Association of Bacteroides concentration with mucosal inflammation has been reported, which is capable of inducing colitis in case the barrier function of colonic enterocytes is damaged [65]. In this work, relative Bacteroides abundances were elevated in IUGR suckling lambs. Elevated intestinal levels of SCFAs produce an effect on the epithelial intactness and other regional physiology of the gut mucosa [66]. Butyrate is generated by stodgy carbohydrate fermentation in the gut microbiota, which is a major outstanding SCFA [67]. The intestinal epithelial cell proliferation and differentiation can be facilitated by butyrate, which also regulates the intestinal barrier function and produces anti-inflammatory and antioxidant activities [54]. As an important genus of butyrate-generating bacteria, Clostridium plays a crucial role in gut homeostasis maintenance [68]. The synthesis of butyrate from lactate and acetate was achievable via the acetate-CoA transferase and butyryl kinase pathways [69]. Streptococcus, on the other hand, could generate acetate via the Wood–Ljungdahl pathway, whose interaction with other bacteria resulted in butyrate formation [70]. According to the findings of the present work, the colonic digesta levels of acetate and butyrate were elevated in IUGR lambs following Arg or NCG supplementation, which agreed with the elevations in the relative Streptococcus and Clostridium abundances. Specifically, the colonic relative Staphylococcus abundance declined sharply after supplementation with Arg or NCG. The S. aureus enterotoxin B, which is secreted by S. aureus of the Staphylococcus genus, is capable of generating a potent activator of immunity, which can interrupt barrier function [71]. The Arg or NCG supplementation can improve the barrier functions by reducing the abundance of Staphylococcus. The remarkably elevated relative Clostridium and Lactobacillus abundances in the colonic mucosa by Arg or NCG supplementation are another contributor. The majority of bacteria in these two genera are beneficial and exert vital roles in resisting gut pathogens and sustaining the immunity and homeostasis of the hosts [72]. The above discussion supports the assumption that the altered colonic epithelial microbiota is the mediator of improved mucosal congenital immune homeostasis, OS, and barrier function in IUGR suckling lambs supplemented with Arg or NCG.

## 5. Conclusions

Our findings suggest that the dietary supplementation with Arg or NCG could efficiently alleviate colonic inflammation, OS, and barrier function impairment and thus make the colon more intact for suckling lambs with IUGR. There is a possible close association between the intestinal health maintenance with Arg or NCG and the elevations in relative Firmicutes, Streptococcus, Lactobacillus, and Clostridium abundances and butyrate levels together with the declined abundances of Bacteroides, Proteobacteria, Staphylococcus, and Escherichia. This work contributes to a better understanding of the crosstalk among microbiota, immunity, OS, and barrier function and emphasizes the potential of Arg or NCG in health enhancement as feed additives in the early life of ruminants. Further investigations should highlight the pathways of immune system responses to IUGR lambs supplied with Arg or NCG.

## Figures and Tables

**Table 1:** Effects of dietary Arg or NCG supplementation on the colonic permeability in the colon of IUGR suckling lambs ^1^.

Item	CON ^2^	IUGR ^3^	IUGR + Arg ^4^	IUGR + NCG ^5^	SEM	*p*-Value
TER, Ω·cm^2^	17.2 ^a^	9.48 ^c^	12.9 ^b^	13.1 ^b^	1.28	0.008
FD4, μg·cm^−2^·h^−1^	14.5 ^c^	23.1 ^a^	17.4 ^b^	18.3 ^b^	1.37	0.027

FD4 = fluorescein isothiocyanate dextran 4 kDa; TER = transepithelial electrical resistance. ^a,b,c^ Mean values within a row with different superscript letters were significantly different (*p* < 0.05). ^1^ Mean values with their standard errors of the mean (SEM), *n* = 12 in each group. ^2^ CON: normal birth weight group given a control diet. ^3^ IUGR: intrauterine-growth-retarded group given a control diet. ^4^ IUGR + Arg: IUGR supplemented with 1% L-arginine. ^5^ IUGR + NCG: IUGR supplemented with 0.1% N-carbamylglutamate.

**Table 2:** Effects of dietary Arg or NCG supplementation on the cytokine concentrations in the colonic mucosa of IUGR suckling lambs ^1^.

Item	CON ^2^	IUGR ^3^	IUGR + Arg ^4^	IUGR + NCG ^5^	SEM	*p*-Value
TNF-α, ng g^−1^ protein	9.11 ^c^	17.89 ^a^	13.16 ^b^	12.98 ^b^	1.062	0.009
IL-1β, ng g^−1^ protein	14.12 ^c^	22.26 ^a^	17.89 ^b^	18.01 ^b^	1.291	0.012
IL-6, ng g^−1^ protein	10.47 ^c^	19.89 ^a^	14.68 ^b^	14.72 ^b^	0.876	0.018

IL = interleukin; TNF-α = tumor necrosis factor α. ^a,b,c^ Mean values within a row with different superscript letters were significantly different (*p* < 0.05). ^1^ Mean values with their standard errors of the mean (SEM), *n* = 12 in each group. ^2^ CON: normal birth weight group given a control diet. ^3^ IUGR: intrauterine-growth-retarded group given a control diet. ^4^ IUGR + Arg: IUGR supplemented with 1% L-arginine. ^5^ IUGR + NCG: IUGR supplemented with 0.1% N-carbamylglutamate.

**Table 3:** Effect of dietary Arg or NCG supplementation on VFAs and LPS concentrations in the colonic digesta of IUGR suckling lambs ^1^.

Item	CON ^2^	IUGR ^3^	IUGR + Arg ^4^	IUGR + NCG ^5^	SEM	*p*-Value
LPS, ng/mL	9.86 ^c^	21.45 ^a^	15.13 ^b^	14.99 ^b^	1.513	0.009
Acetate, μmol/g	62.96 ^a^	38.19 ^c^	47.43 ^b^	50.11 ^b^	2.872	0.019
Propionate, μmol/g	17.16 ^a^	10.42 ^c^	13.89 ^b^	14.02 ^b^	1.461	0.008
Butyrate, μmol/g	4.68 ^a^	2.68 ^c^	3.59 ^b^	3.64 ^b^	0.794	0.021
Isobutyrate, μmol/g	6.48 ^a^	2.59 ^c^	4.08 ^b^	4.21 ^b^	0.287	0.005
Valerate, μmol/g	1.69	1.72	1.67	1.70	0.208	0.098
Isovalerate, μmol/g	0.78	0.81	0.77	0.80	0.083	0.207

LPS = lipopolysaccharide. ^a,b,c^ Mean values within a row with different superscript letters were significantly different (*p* < 0.05). ^1^ Mean values with their standard errors of the mean (SEM), *n* = 12 in each group. ^2^ CON: normal birth weight group given a control diet. ^3^ IUGR: intrauterine-growth-retarded group given a control diet. ^4^ IUGR + Arg: IUGR supplemented with 1% L-arginine. ^5^ IUGR + NCG: IUGR supplemented with 0.1% N-carbamylglutamate.

**Table 4:** Effects of dietary Arg or NCG supplementation on the mRNA abundance of genes in the colon of IUGR suckling lambs ^1^.

Item	CON ^2^	IUGR ^3^	IUGR + Arg ^4^	IUGR + NCG ^5^	SEM	*p*-Value
Barrier function-related genes						
*ZO-1*	1.00 ^a^	0.52 ^c^	0.69 ^b^	0.92 ^a^	0.121	0.009
*Occludin*	1.00 ^a^	0.61 ^c^	0.82 ^b^	0.79 ^b^	0.152	0.006
*Claudin-1*	1.00 ^a^	0.48 ^c^	0.69 ^b^	0.71 ^b^	0.093	0.012
Immune function-related genes						
*MyD88*	1.00 ^c^	1.63 ^a^	1.31 ^b^	1.35 ^b^	0.078	0.009
*TLR-4*	1.00 ^b^	1.33 ^a^	1.19 ^ab^	1.14 ^ab^	0.098	0.023
*NF-κB*	1.00 ^c^	1.65 ^a^	1.30 ^b^	1.27 ^b^	0.162	0.019
*TNF-α*	1.00 ^c^	1.70 ^a^	1.42 ^b^	1.39 ^b^	0.094	0.008
*IL-1β*	1.00 ^b^	1.58 ^a^	1.05 ^b^	0.97 ^b^	0.069	0.013
*IL-6*	1.00 ^c^	1.69 ^a^	1.36 ^b^	1.37 ^b^	0.128	0.006

MyD88 = myeloid differentiation factor 88; TLR = toll-like receptor; IL = interleukin, TNF-α = tumor necrosis factor α; NF-κB = nuclear factor kappa-B; ZO-1 = zonula occludens-1. ^a,b,c^ Mean values within a row with different superscript letters were significantly different (*p* < 0.05). ^1^ Mean values with their standard errors of the mean (SEM), *n* = 12 in each group. ^2^ CON: normal birth weight group given a control diet. ^3^ IUGR: intrauterine-growth-retarded group given a control diet. ^4^ IUGR + Arg: IUGR supplemented with 1% L-arginine. ^5^ IUGR + NCG: IUGR supplemented with 0.1% N-carbamylglutamate.

**Table 5:** Effects of dietary Arg or NCG supplementation on the average richness and diversity of the colonic mucosal bacterial community at the 3% dissimilarity level in the IUGR suckling lambs ^1^.

Item	CON ^2^	IUGR ^3^	IUGR + Arg ^4^	IUGR + NCG ^5^	SEM	*p*-Value
OTUs	1198 ^a^	639 ^c^	895 ^b^	903 ^b^	34.2	0.007
ACE	1386 ^a^	692 ^c^	912 ^b^	957 ^b^	40.8	0.005
Chao 1	1279 ^a^	603 ^c^	903 ^b^	893 ^b^	39.3	0.010
Shannon index	5.12 ^a^	3.25 ^c^	4.02 ^b^	4.13 ^b^	0.277	0.009
Simpson index	0.94	0.90	0.95	0.91	0.052	0.085

ACE = abundance-based coverage estimator; OTUs = operational taxonomic units. ^a,b,c^ Mean values within a row with different superscript letters were significantly different (*p* < 0.05). ^1^ Mean values with their standard errors of the mean (SEM), *n* = 12 in each group. ^2^ CON: normal birth weight group given a control diet. ^3^ IUGR: intrauterine-growth-retarded group given a control diet. ^4^ IUGR + Arg: IUGR supplemented with 1% L-arginine. ^5^ IUGR + NCG: IUGR supplemented with 0.1% N-carbamylglutamate.

**Table 6:** Effects of dietary Arg or NCG supplementation on the average relative abundance of phylum (% of total sequences) in the colonic mucosa of the IUGR suckling lambs ^1^.

Item	CON ^2^	IUGR ^3^	IUGR + Arg ^4^	IUGR + NCG ^5^	SEM	*p*-Value
*Acidobacteria*	1.35	1.32	1.36	1.33	0.228	0.118
*Actinobacteria*	4.27	4.19	4.63	4.81	0.392	0.094
*Bacteroidetes*	9.12 ^c^	16.47 ^a^	12.68 ^b^	13.03 ^b^	1.121	0.009
*Chloroflexi*	1.24	1.17	1.23	1.19	0.327	0.103
*Cyanobacteria*	4.13	4.32	4.09	4.26	0.183	0.079
*Firmicutes*	55.34 ^a^	31.19 ^c^	43.12 ^b^	42.32 ^b^	3.476	0.005
*Proteobacteria*	4.78 ^c^	9.03 ^a^	6.83 ^b^	7.02 ^b^	1.032	0.013

^a,b,c^ Mean values within a row with different superscript letters were significantly different (*p* < 0.05). ^1^ Mean values with their standard errors of the mean (SEM), *n* = 12 in each group. ^2^ CON: normal birth weight group given a control diet. ^3^ IUGR: intrauterine-growth-retarded group given a control diet. ^4^ IUGR + Arg: IUGR supplemented with 1% L-arginine. ^5^ IUGR + NCG: IUGR supplemented with 0.1% N-carbamylglutamate.

**Table 7:** Effects of dietary Arg or NCG supplementation on average relative abundance of genus (% of total sequences) in colonic mucosa of the IUGR suckling lambs ^1^.

Phylum	Genus	CON ^2^	IUGR ^3^	IUGR + Arg ^4^	IUGR + NCG ^5^	SEM	*p*-Value
*Actinobacteria*	*Actinomyces* *Bifidobacterium* *Rothia*	1.56 0.67 0.12	1.64 0.72 0.13	1.62 0.66 0.11	1.59 0.71 0.09	0.287 0.182 0.047	0.077 0.126 0.083
*Bacteroidetes*	*Bacteroides* *Prevotella*	0.18 ^c^ 1.26	0.36 ^a^ 1.31	0.29 ^b^ 1.29	0.25 ^b^ 1.30	0.083 0.152	0.007 0.208
*Firmicutes*	*Blautia* *Butyrivibrio* *Clostridium* *Lactobacillus* *Ruminococcus* *Staphylococcus* *Streptococcus* *Veillonella*	0.88 2.11 0.89 ^a^ 2.01 ^a^ 1.68 1.04 ^c^ 2.21 ^a^ 0.31	1.03 1.98 0.46 ^c^ 1.35 ^c^ 1.73 3.12 ^a^ 1.28 ^c^ 0.29	0.97 1.99 0.62 ^b^ 1.67 ^b^ 1.65 1.94 ^b^ 1.68 ^b^ 0.34	0.91 2.06 0.70 ^b^ 1.72 ^b^ 1.72 1.86 ^b^ 1.71 ^b^ 0.32	0.121 0.757 0.124 0.272 0.321 0.537 0.366 0.082	0.097 0.162 0.008 0.013 0.095 0.008 0.006 0.203
*Proteobacteria*	*Escherichia*	0.78 ^c^	1.36 ^a^	0.97 ^b^	1.02 ^b^	0.321	0.019
	*Pseudomonas*	1.03 ^c^	1.67 ^a^	1.43 ^b^	1.39 ^b^	0.286	0.007

^a,b,c^ Mean values within a row with different superscript letters were significantly different (*p* < 0.05). ^1^ Mean values with their standard errors of the mean (SEM), *n* = 12 in each group. ^2^ CON: normal birth weight group given a control diet. ^3^ IUGR: intrauterine-growth-retarded group given a control diet. ^4^ IUGR + Arg: IUGR supplemented with 1% L-arginine. ^5^ IUGR + NCG: IUGR supplemented with 0.1% N-carbamylglutamate.

## Data Availability

All data relevant to the study are included in the article or uploaded as supplementary information. Data are available on reasonable request. Data generated and analyzed during this study are available from the corresponding author on reasonable request.

## Supplementary Materials

The following supporting information can be downloaded at: https://www.mdpi.com/article/10.3390/antiox11112251/s1, Table S1: Ingredient and nutrient composition of milk replacer; Table S2: Primer sequences used in the real-time PCR; Table S3: Effect of dietary Arg or NCG supplementation on oxidative status and mitochondrial ROS production in the colon of IUGR suckling lambs.
