# Supplementary material for: Dietary L-Arginine or N-Carbamylglutamate Alleviates Colonic Barrier Injury, Oxidative Stress, and Inflammation by Modulation of Intestinal Microbiota in Intrauterine Growth-Retarded Suckling Lambs
Source: Antioxidants (Basel). 2022 Nov 15;11(11):2251. doi: 10.3390/antiox11112251 (PMC9687183; doi:10.3390/antiox11112251)
Supplement: Supplementary file 1 [file antioxidants-11-02251-s001.zip › antioxidants-1960872-supplementary.pdf]

Supplementary Table S1 Ingredient and nutrient composition of milk replacer (dry-matter basis, %)[1].

Item	Milk replacer
Ingredients	
Whey protein concentrate, 34% CP	30.00
Milk fat powder, 11% CP	35.00
Whole milk powder	20.00
a-Casein	5.00
Glucose	6.00
Vitamin and mineral pre-mix [2]	4.00
Nutrient content (analyzed)[3]	
GE, MJ/kg	19.36
CP, %	23.77
EE, %	15.65
Ash, %	7.64
Lysine, %	1.53
Methionine, %	0.41
Threonine, %	0.87
L-arginine, %	0.63
Ca, %	0.99
TP, %	0.73

[1]DM= dry matter; GE= gross energy; CP= crude protein; EE= ether extract; Ca=

calcium; TP= total phosphorus.

[2]Main contents of the pre-mixed mixture (per kg of the pre-mixed mixture): Cu (as $CuSO_4 \cdot 5H_2O$) 600 mg; Mn ($MnSO_4 \cdot H_2O$) 315 mg; Fe ($FeSO_4 \cdot 7H_2O$) 8400 mg; Zn ($ZnSO_4 \cdot 7H_2O$) 12500 mg; Se (as Na_2SeO_3) 17 mg; vitamin A 55000 IU; vitamin E 400 IU; vitamin D 5500 IU; vitamin K 12.5 mg; biotin 2 mg; folacin 7.5 mg; choline 15 mg; riboflavin 100 mg; vitamin B_6 175 mg; thiamin 317.5 mg; and vitamin B_{12} 500 mg.

[3] Nutrient levels are all measured values.

Supplementary Table S2 Primer sequences used in the real-time PCR[1].

Gene	Sequence (5'–3')	GenBank accession number
Barrier function related genes		
ZO-1	F：CGACCAGATCCTCAGGGTAA	XM_015101949.1
	R：AATCACCCACATCGGATTCT	
Occludin	F：GTTCGACCAATGCTCTCTCAG	XM_015101256.1
	R：CAGCTCCCATTAAGGTTCCA	
Claudin-1	F: CACCCTTGGCATGAAGTGTA	NM_001185016.1
	R: AGCCAATGAAGAGAGCCTGA	
Immune function-related genes		
MyD88	F: GCGAGGACGTGCTGATGGAAC	NM_001166183.1
	R: GATGCCTCCTGCTGCTGCTTC	
TLR-4	F: TGTGAAGGACATGCCAGTGCTTG	NM_001135930.1
	R：TGACAACCGACACGCTGATGATC	
NF-κB	F：ATTCAGCCCTTTGCCCATCT	EF121765.1
	R：ATGGGATGTCAGTGGCGTTA	
TNF-α	F: GGGAACACAGACAGAGGGGACA	EF446377.1
	R：CCTGCGAGTAGATGAGGTAAAG	
IL-1β	F: CGTCTTCCTGGGACGTTTTAG	NM_001009465.2
	R: CTGCGTATGGCTTCTTTAGGG	
IL-6	F: GCAGACTACTTCTGACCACTCCA	NM_001009392.1
	R: TTTTCACACTCGTCATTCTTCTCAC	

β-actin	F：ATGAGGCTCAGAGCAAGAGA	NM_001009784.3
	R：ACACGCAGCTCGTTGTAGAA	

[1]MyD88= myeloid differentiation factor 88; TLR= toll-like receptor; IL= interleukin, TNF-α= tumor necrosis factor α; NF-κB= nuclear factor kappa-B; ZO-1= zonula occludens-1.

Supplementary Table S3 Effect of dietary Arg or NCG supplementation on oxidative status and mitochondrial ROS production in the colon of IUGR suckling lambs[1].

Item[2]	CON	IUGR	IUGR + Arg	IUGR + NCG	SEM	P value
ROS production, fold change	1.00[c]	2.29[a]	1.58[b]	1.63[b]	0.112	0.008
T-AOC, U/mg protein	1.99[a]	1.21[c]	1.57[b]	1.51[b]	0.141	0.023
MDA, nmol/mg protein	0.35[c]	0.52[a]	0.47[b]	0.44[b]	0.038	0.009
GSH-Px, U/mg protein	15.3[a]	7.37[c]	10.2[b]	11.0[b]	1.03	0.012
SOD, U/mg protein	111[a]	57.9[c]	84.3[b]	109[a]	5.39	0.005
Protein carbonyl, nmol/mg protein	1.87[c]	2.76[a]	2.21[b]	2.19[b]	0.132	0.017
GR, U/g protein	4.13[c]	6.13[a]	4.98[b]	5.02[b]	0.356	0.028
GSH, nmol/mg protein	1.58[a]	0.81[c]	1.14[b]	1.19[b]	0.102	0.005
GSSG, nmol/mg protein	0.10	0.12	0.11	0.13	0.031	0.089
GSH/GSSG	15.8[a]	6.75[c]	10.4[b]	9.16[b]	1.149	0.008
H_2O_2, mmol/g of protein	11.2[c]	18.3[a]	14.4[b]	14.6[b]	1.67	0.016

[a,b,c] Within a row, mean values without a common letter differ ($P < 0.05$).

[1]Mean values with their SEM (n = 12 per group). These results have been published in our previous paper (Zhang et al., 2022).

[2]CON = the normal birth weight group given a control diet; GSH-Px = glutathione peroxidase; GR = glutathione reductase; GSH = reduced glutathione; GSSG = oxidized glutathione; H_2O_2 = hydrogen peroxide; IUGR = the intrauterine-growth-retarded group given a control diet; IUGR + Arg = IUGR supplemented with 1% L-arginine; IUGR + NCG = IUGR supplemented with 0.1% N-carbamylglutamate; MDA = malondialdehyde; ROS = reactive oxygen species; SOD = superoxide dismutase; T-

AOC = total antioxidant capacity.
